# Trust, validation, and accountability: implementing uses of generative artificial intelligence into Health Technology Assessment practice

**DOI:** 10.1017/S0266462326103638

**Published:** 2026-03-05

**Authors:** Rachael Fleurence, Jagpreet Chhatwal

**Affiliations:** 1 Apodeixis Strategies LLC, USA; 2 Center for Health Technology Assessment, Mass General Brigham, Harvard Medical School, USA

**Keywords:** GenAI, artificial intelligence, systematic literature reviews, HTA, evidence synthesis, generative artificial intelligence, methods

## Abstract

The 2025 HTAi Global Policy Forum (GPF) report offers a timely and thoughtful synthesis of the opportunities and challenges associated with the use of artificial intelligence (AI), and particularly generative AI (GenAI), in Health Technology Assessment (HTA) (1). Its emphasis on trust, human agency, and risk-based approaches reflects both the maturity of the discussion within the HTA community and a shared recognition that technical capability alone is insufficient for responsible adoption. The report succeeds in articulating a common set of principles and a broadly aligned vision across HTA bodies, life sciences, and other interest holders.

The 2025 HTAi Global Policy Forum (GPF) report offers a timely and thoughtful synthesis of the opportunities and challenges associated with the use of artificial intelligence (AI), and particularly generative AI (GenAI), in Health Technology Assessment (HTA) (1). Its emphasis on trust, human agency, and risk-based approaches reflects both the maturity of the discussion within the HTA community and a shared recognition that technical capability alone is insufficient for responsible adoption. The report succeeds in articulating a common set of principles and a broadly aligned vision across HTA bodies, life sciences, and other interest holders.

The central question now is how these principles translate into operational practice. As GenAI tools move from experimentation toward potential routine use, HTA organizations face a practical challenge: identifying where GenAI meaningfully improves and accelerates HTA processes, where it introduces new risks, and how its use should be evaluated, documented, and governed (2). Drawing on recent empirical evidence and experience from evidence synthesis workflows, this response aims to complement the GPF’s strategic framing by highlighting lessons from early applications and suggesting concrete directions for the next phase of integration.

## GenAI as augmentation, not replacement

A consistent message emerging from early applications of GenAI in HTA-related workflows is that value is highly task-dependent. At this stage, GenAI performs best when used to augment clearly defined, bounded tasks, rather than to replace complex, end-to-end analytic processes. This observation aligns well with the GPF’s emphasis on risk-based adoption: not all HTA activities carry the same consequences if errors occur, and not all tasks require the same level of human judgment or contextual interpretation.

Systematic literature reviews (SLRs) provide a particularly instructive example. SLRs are foundational to HTA, but they are also resource-intensive, slow, and vulnerable to human error. Traditionally, dual independent review has been treated as the methodological gold standard, yet human reviewers are not infallible. Errors in screening, data extraction, and even analytic decisions are possible. Recent work illustrates this point clearly. In a large-scale evaluation of an agentic, GenAI-enabled SLR workflow, investigators were able to reproduce and update an entire issue of Cochrane reviews in 2 days and identified several instances in which eligible studies had been missed or data had been inconsistently extracted in the original reviews (3). Importantly, this does not represent a failure of Cochrane methodology; rather, it highlights an uncomfortable but well-known truth for HTA: human-led processes are themselves prone to error, even when conducted rigorously and in good faith. One illustrative example comes from a nutrition review, where the AI-assisted workflow identified five additional eligible studies beyond those included in the original Cochrane review (3). Incorporating this additional evidence materially changed the conclusions: whereas the original review found no clear effect of preoperative immune-enhancing nutrition on hospital length of stay, the updated analysis showed an average reduction of ~1 day compared with usual care. Notably, this revised finding was consistent across multiple corrected and updated analyses conducted by the authors, underscoring that AI-supported workflows, when carefully validated, can surface clinically meaningful evidence that may otherwise be overlooked. Seen in this light, GenAI should not be framed solely as a source of new risk. Properly designed and validated, it can also serve as a mechanism for quality improvement, reproducibility checks, and error detection within existing HTA workflows.

## What the evidence shows – and what it does not

Empirical evaluations of GenAI in evidence synthesis consistently show that performance varies substantially by SLR task, cautioning against sweeping claims of overall readiness and pointing instead to the need for task-specific evaluation (4). The strongest and most consistent evidence comes from early, well-defined steps of the systematic review workflow, particularly title and abstract screening and structured data extraction. In these settings, several studies report that GenAI systems can match or exceed the sensitivity of traditional dual human review while maintaining comparable specificity. For example, in a large-scale evaluation of an agentic GenAI-enabled workflow, Cao et al. reported substantially higher sensitivity for AI-assisted screening than for dual human reviewers (96.7 percent vs. 81.7 percent), with nearly identical specificity (97.9 percent vs. 98.1 percent). In the same study, GenAI also achieved higher accuracy in structured data extraction when benchmarked against adjudicated reference standards (93.1 percent vs. 79.7 percent for human reviewers) (3).

Complementary findings are reported by Zhang et al., who evaluated a GenAI pipeline across 100 published systematic reviews encompassing more than 2,200 clinical studies (5). In pilot human-AI collaboration experiments, AI-assisted workflows improved recall during screening by over 70 percent while reducing screening time by more than 40 percent. For data extraction, accuracy increased by ~24 percent, alongside time reductions exceeding 60 percent. In another study, Tran et al. evaluated a general-purpose language model as a second reviewer for title and abstract screening and found that it identified nearly all eligible studies (over 90 percent) but at the cost of flagging a large number of nonrelevant citations, substantially increasing the need for human reconciliation (6). Used as a triage tool, the model could reduce the number of citations requiring manual screening by more than half while missing a few or no eligible studies at the full-text stage, highlighting both the efficiency gains and workload trade-offs of assistive GenAI use in screening (6).

Together, these results suggest that GenAI can meaningfully enhance both efficiency and accuracy for constrained, well-specified tasks within evidence synthesis workflows. However, these findings require careful interpretation. High performance has been observed primarily in settings where models are tightly constrained – provided with explicit eligibility criteria, predefined extraction fields, and structured prompts. In contrast, attempts to deploy GenAI for fully autonomous, end-to-end systematic reviews, spanning question formulation through synthesis, have yielded far more variable and often underwhelming results. Such approaches remain difficult to validate, lack transparency, and are poorly aligned with HTA norms around reproducibility and methodological defensibility (4).

The potential role of GenAI in HTA extends beyond systematic reviews. Emerging use cases include drafting and harmonizing submission documents across jurisdictions, generating plain-language summaries for patients, synthesizing public comments, supporting health economic model documentation, and assisting in horizon scanning or early evidence mapping. In principle, GenAI may also facilitate “living HTA” models by enabling more continuous updating of evidence and analytic components. However, as with SLRs, the degree of readiness varies by task.

This distinction is critical for HTA. HTA places particular emphasis on evidence that can be transparently assessed, independently scrutinized, and methodologically justified. The emerging lesson from the current evidence base is therefore not that GenAI is uniformly “ready” or “not ready” for HTA, but that its readiness is task-dependent – and that performance claims must be explicitly tied to specific, well-defined use cases rather than generalized across the entire systematic review process.

## Trust is built through validation and reporting

Trust emerged as a central theme in the GPF discussions, reflecting broad agreement that the responsible use of GenAI in HTA will depend not only on intent but on demonstrable validation and transparent reporting. While the Forum appropriately emphasizes disclosure, human oversight, and risk-based approaches, operational guidance on *how* trust should be established in practice remains limited. In particular, there is a need for greater clarity on what information should be reported when GenAI is used in HTA activities and how the credibility of AI-assisted outputs should be assessed across different tasks and levels of risk. Recent work by the ISPOR Working Group on Generative AI, including the ELEVATE-GenAI reporting guidelines, helps bridge this gap by translating high-level principles into task-specific, use-case-driven reporting expectations aligned with established HTA norms (7). Rather than treating trust as a binary property of a model, ELEVATE-GenAI emphasizes that trust must be earned at the level of the task, with validation approaches tailored to the nature and consequences of the application. For structured tasks, such as title and abstract screening or data extraction, quantitative performance metrics and benchmarking against reference standards may be appropriate, whereas a more qualitative assessment is required for generative tasks such as narrative synthesis or drafting. Importantly, the framework makes human agency explicit by requiring authors to document where and how human oversight occurs, how outputs are verified, and how disagreements between human judgment and model outputs are resolved. Early applications of the framework also reveal uneven methodological maturity across evaluation domains: while accuracy, reproducibility, and factual verification are increasingly well addressed, domains such as bias, calibration, and data governance remain less developed. This unevenness helps explain the cautious and variable adoption of GenAI across HTA organizations and underscores that trust in AI-assisted HTA outputs is built through validation, reporting, and shared methodological expectations, rather than assumed a priori.

## Human agency as accountability

The GPF’s emphasis on human agency is both timely and essential. Yet “human-in-the-loop” is often invoked without sufficient clarity about what responsibilities humans actually retain. In HTA, human agency must extend beyond nominal oversight to include accountability for methodological choices, validation of outputs, and final decision-making.

This has practical implications. As GenAI tools become more capable, there is a risk that responsibility becomes diffused: reviewers rely on models, organizations rely on vendors, and accountability becomes unclear. HTA bodies will need to explicitly define roles, competencies, and escalation pathways when GenAI is used – particularly for high-impact decisions.

Responsible adoption is therefore not solely a technical challenge; it is also a capacity-building challenge. Investment in AI literacy, methodological training, and governance structures will be as important as the tools themselves.

## Why adoption has been appropriately slow

The GPF report notes variability in digital maturity and GenAI uptake across HTA organizations. This unevenness is sometimes characterized as resistance or lag, but it can also be understood as a rational response to unresolved questions about validation, governance, and legal responsibility.

Despite increasing technical capability, significant barriers continue to limit the widespread implementation of GenAI in HTA practice. Many of these barriers are legal and institutional rather than methodological. A central concern is data governance: what information can be uploaded into AI systems, under what conditions, and for what purposes. HTA workflows often involve unpublished clinical data, confidential pricing information, proprietary models, and commercially sensitive submissions. In many jurisdictions, it remains unclear whether uploading such materials into third-party AI tools – particularly cloud-based platforms – constitutes data sharing or disclosure under privacy, intellectual property, or procurement laws. Therefore, transparent data governance policies will be essential if GenAI is to move from isolated pilots to sustained, system-level integration within HTA.

HTA operates under public accountability and often statutory mandates. Moving cautiously in the absence of clear standards is not a failure of innovation; it is an expression of institutional responsibility. The risk for the field is not slow adoption per se, but fragmented adoption – where tools are used inconsistently, without shared benchmarks or learning.

## Moving forward: from principles to methods

The GPF’s proposed next steps – including continued dialogue, communities of practice, and further methodological work – are well aligned with the needs of the field. A critical priority now is to advance method maturity in parallel with experimentation. This includes clearer guidance on task-level validation, minimum reporting standards, and appropriate use cases for GenAI within HTA processes.

If GenAI is integrated thoughtfully – as an assistive technology, evaluated rigorously, and reported transparently – it has the potential not only to increase efficiency, but also to strengthen the reliability and reproducibility of HTA itself. The challenge ahead is not whether to adopt GenAI, but how to do so in a way that reinforces, rather than undermines, the values on which HTA depends.
